# Paraneoplastic Pemphigus/Paraneoplastic Autoimmune Multiorgan Syndrome Associated With Castleman Disease: Multidisciplinary Management of a Rare Life‐Threatening Syndrome

**DOI:** 10.1002/ccr3.73105

**Published:** 2026-07-08

**Authors:** Ayaan Sohail, Jessica Evans, Mika Tabata

**Affiliations:** ^1^ John P. And Kathrine G. McGovern Medical School, University of Texas Health Science Center at Houston Houston Texas USA; ^2^ Department of Dermatology The University of Texas MD Anderson Cancer Center Houston Texas USA

**Keywords:** bronchiolitis obliterans, Castleman disease, envoplakin antibodies, multidisciplinary management, paraneoplastic autoimmune multiorgan syndrome, paraneoplastic pemphigus

## Abstract

Refractory oral erosions or polymorphous eruptions should prompt evaluation for paraneoplastic pemphigus, particularly when associated with mediastinal masses. Early tumor excision, multidisciplinary care, and vigilant monitoring for bronchiolitis obliterans are essential to improve outcomes in Castleman disease–associated paraneoplastic autoimmune multiorgan syndrome.

## 1 Introduction

Paraneoplastic pemphigus (PNP), formally described in 1990 by Grant Anhalt, identified five patients with underlying neoplasms who developed painful mucosal ulcerations and polymorphous skin lesions with a unique autoantibody profile, and proposed the term “paraneoplastic pemphigus” to distinguish this condition from classical pemphigus vulgaris and pemphigus foliaceus. PNP is characterized by painful mucocutaneous blistering, erosive stomatitis, and polymorphous skin eruptions. The term paraneoplastic autoimmune multiorgan syndrome (PAMS) was coined in 2001 to convey the multiorgan involvement associated with mucocutaneous disease and broader immunologic abnormalities. Mortality in PNP/PAMS is as high as 80%. Castleman disease (CD) is a rare, heterogenous lymphoproliferative disorder associated with several conditions, including PNP/PAMS. PNP/PAMS is most frequently linked to unicentric CD, particularly the hyaline vascular subtype, and is mediated by autoantibodies targeting plakin family proteins and desmosomal cadherins, with both humoral and cell‐mediated immune mechanisms implicated in its pathogenesis [1, 2, 3, 4].

The clinical course of PNP/PAMS in CD is frequently complicated by bronchiolitis obliterans, a progressive and often fatal pulmonary manifestation, and may also be associated with myasthenia gravis and follicular dendritic cell sarcoma [5]. Complete surgical resection of unicentric Castleman tumors can lead to resolution of mucocutaneous lesions and reduction in autoantibody titers, but pulmonary complications may persist and are a major determinant of prognosis. The overall survival is significantly reduced in patients with PNP/PAMS associated CD, especially in those with bronchiolitis obliterans or incomplete tumor resection [6].

We present a case of PNP/PAMS associated with unicentric CD complicated by life‐threatening bronchiolitis obliterans, emphasizing the diagnostic challenges and therapeutic limitations of this condition. This report highlights the critical importance of early recognition and multidisciplinary management in mitigating mortality from this aggressive syndrome.

## 2 Case Examination

**FIGURE 1:**
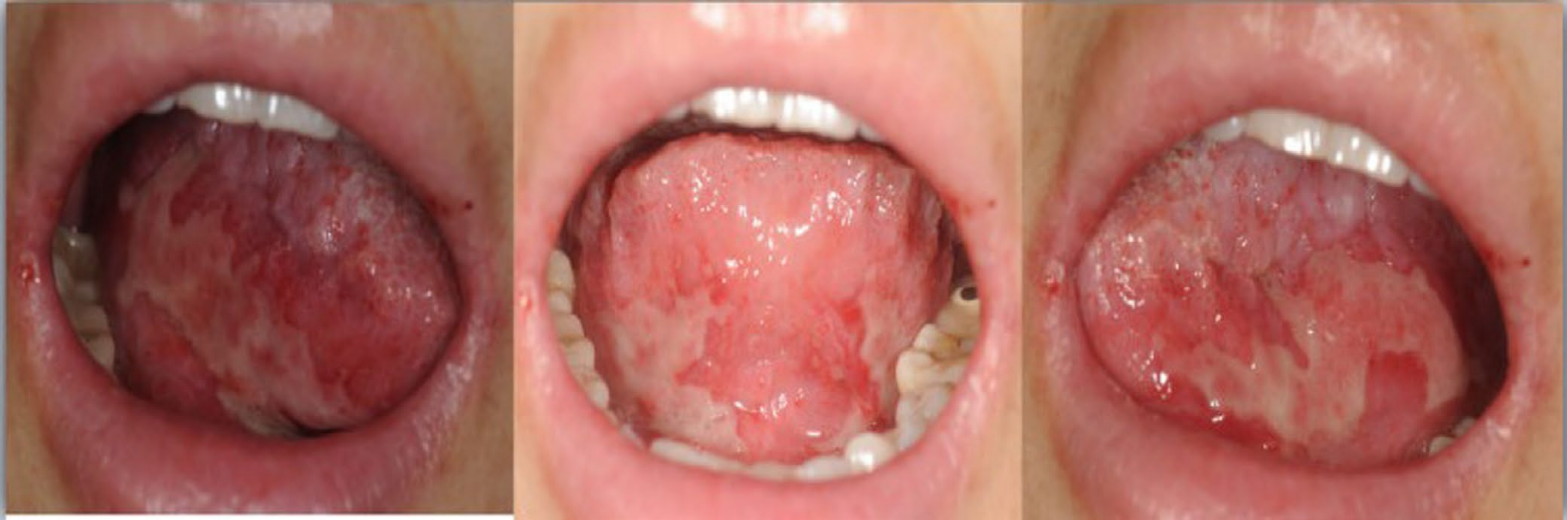
Painful erosions and ulcerations of the tongue and buccal mucosa with erythematous bases.

**FIGURE 2:**
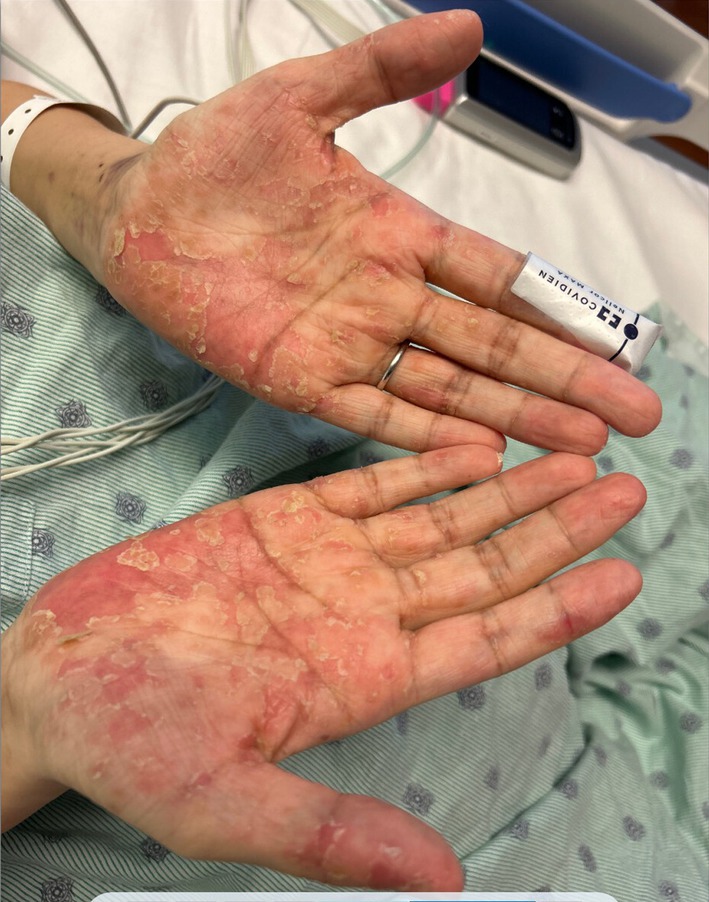
Dusky erythematous patches and early bullous changes on the palms.

A previously healthy 39‐year‐old woman presented with painful oral erosions (Figure 1), erythematous gingiva, and bullous plaques on the palms (Figure 2). Her symptoms had persisted for several months despite multiple topical therapies, including nystatin and dexamethasone rinses prescribed by her primary care physician and oral surgeon. The patient did not report constitutional symptoms, including fever, night sweats, or weight loss, and physical examination revealed no evidence of hepatosplenomegaly. Given the refractory nature of her mucosal disease, a biopsy of the buccal mucosa was performed.

Histopathologic examination demonstrated focal acantholysis and areas of lichenoid interface mucositis, with scattered apoptotic and necrotic keratinocytes within the basal layer. The lamina propria contained a dense, mixed inflammatory infiltrate composed predominantly of lymphocytes and plasma cells with scattered eosinophils. Direct immunofluorescence and a paraneoplastic antibody panel revealed circulating IgG with positive envoplakin antibodies, confirming the diagnosis of PNP.

Physical examination revealed widespread painful erosions of the oral mucosa (Figure 1), erythematous gingiva, and dusky erythematous patches with early bullous changes on the palms (Figure 2). Computed tomography (CT) scan of the chest demonstrated a 7.2 cm anterior mediastinal mass. Surgical excision of the mass revealed unicentric CD, hyaline vascular subtype, without evidence of systemic nodal involvement. Initial laboratory evaluation demonstrated a normocytic anemia with hemoglobin of 10.0 g/dL (subsequently 9.8 g/dL), a normal total leukocyte count (7.9–7.1 K/μL) with marked neutrophilia (88.9%) and relative lymphopenia (absolute lymphocyte count 0.32–0.34 K/μL), and normal platelet counts (189–190 K/μL). Renal function was preserved, with blood urea nitrogen of 12 mg/dL and creatinine of 0.23–0.24 mg/dL. Electrolytes were notable for mild hypochloremia (93–94 mmol/L) and elevated bicarbonate levels (39–40 mmol/L). Inflammatory markers, including erythrocyte sedimentation rate (ESR) and C‐reactive protein (CRP), were obtained as part of the initial evaluation; however, specific values were not available for review.

Following tumor resection, the patient's clinical course was complicated by progressive dyspnea, hypoxemia, and recurrent admissions to the intensive care unit. Pulmonary evaluation confirmed bronchiolitis obliterans. Her oral and cutaneous lesions improved, but despite treatment with high‐dose systemic corticosteroids, intravenous immunoglobulin, and rituximab, her pulmonary status continued to deteriorate, ultimately requiring intubation for acute hypercapnic respiratory failure. Additional complications included cytomegalovirus viremia, profound weight loss, and steroid‐induced insomnia and weakness. Serial antibody testing demonstrated decreasing envoplakin titers following tumor resection and immunotherapy, suggesting partial immunologic response despite limited clinical improvement. Following extubation, she was transferred for lung transplant consideration.

## 3 Differential Diagnosis

The differential diagnosis for this patient's severe mucocutaneous disease initially included pemphigus vulgaris, erythema multiforme, Stevens‐Johnson syndrome, lichen planus, mucous membrane pemphigoid, Behçet disease, graft‐versus‐host disease–like reactions, and infectious etiologies including herpes simplex virus and candidiasis. Given the association of paraneoplastic pemphigus (PNP) with underlying malignancy, lymphoproliferative disorders such as lymphoma, chronic lymphocytic leukemia, thymoma, and plasma cell dyscrasias were also considered. The patient additionally underwent evaluation for multicentric Castleman disease and human herpesvirus‐8 (HHV‐8)–associated disease, both of which were excluded.

Histopathologic evaluation of the buccal mucosa demonstrated focal acantholysis with lichenoid interface mucositis and apoptotic keratinocytes. Direct immunofluorescence and paraneoplastic antibody testing identified circulating IgG autoantibodies with positive envoplakin antibodies, supporting the diagnosis of PNP/PAMS. Computed tomography of the chest revealed a 7.2 cm anterior mediastinal mass, and surgical pathology following excision confirmed unicentric Castleman disease of the hyaline vascular subtype. Pulmonary evaluation, including imaging and respiratory assessment, confirmed bronchiolitis obliterans after the patient developed progressive dyspnea and hypoxemia. Laboratory studies demonstrated normocytic anemia, neutrophilia with relative lymphopenia, and mild electrolyte abnormalities, while inflammatory markers were elevated during the initial workup.

## 4 Conclusion

Future work should identify early indicators of response to treatment to help clinicians balance aggressive treatment with the side effects of extreme immunosuppression. This case highlights the importance of recognizing refractory oral erosions and polymorphous skin lesions as potential paraneoplastic phenomena in the setting of mediastinal masses or unexplained lymphadenopathy. Early identification of PNP/PAMS–associated CD, prompt surgical management, and coordinated multidisciplinary care with oncology, surgery, dermatology, pulmonology, immunology/rheumatology, and possibly otolaryngology, infectious disease, and ophthalmology, are critical to improving outcomes and mitigating irreversible pulmonary decline.

## 5 Discussion

PNP/PAMS associated with CD represents a distinct clinicopathologic entity characterized by severe mucocutaneous involvement and a high risk of life‐threatening complications. Although CD is most commonly seen in children, it can occur in adults as in our case. PNP/PAMS is most frequently linked to the hyaline vascular variant of unicentric CD, as seen in the presented case, although it can also occur in multicentric forms and other histologic subtypes. Pathogenesis involves both humoral and cell‐mediated autoimmunity, with autoantibodies directed against plakin family proteins (envoplakin, periplakin, desmoplakin) and desmosomal cadherins, which are highly diagnostic for PNP in this setting. The neoplastic B cells within Castleman tumors have been shown to generate these pathogenic antibodies, directly implicating the tumor as the source of autoimmunity [7].

Diagnosis requires integration of clinical, histopathologic, and serologic findings. Clinically, patients often present with painful, treatment‐resistant stomatitis and polymorphous cutaneous eruptions. Skin and mucosal biopsies demonstrate acantholysis and lichenoid dermatitis/mucositis often with a lymphoplasmacytic infiltrate. Indirect immunofluorescence (IIF) on rat bladder—positive in 74%–86% of cases with a specificity of 83%–98%—along with immunoblotting or immunoprecipitation assays detecting antibodies against envoplakin, periplakin, and desmoplakin (sensitivity 80%–89%, specificity 94%–100%) remains highly specific for paraneoplastic pemphigus and aids in distinguishing PNP‐associated Castleman disease from classic pemphigus vulgaris or erythema multiforme–like eruptions. Although desmoglein ELISAs may be positive, they lack specificity for PNP and should be interpreted cautiously [8, 9, 10, 11, 12, 13]. Patients should be closely evaluated and monitored for the development of systemic symptoms. Imaging should be performed to evaluate for associated pulmonary involvement.

Management of PNP‐associated CD centers on early tumor resection and immunosuppressive therapy. Although complete excision can attenuate antibody production, immunologic and pulmonary sequelae frequently persist. Adjunctive therapies with systemic corticosteroids, rituximab, intravenous immunoglobulin, and plasma exchange have shown variable success. Lung transplantation may be considered in select cases with severe bronchiolitis obliterans [14]. The presence of PNP/PAMS remains an independent adverse prognostic factor in CD, with mortality rates approaching 90% when bronchiolitis obliterans develops [15, 16, 17].

In our patient's case, surgical removal of the tumor led to a partial serologic response, evidenced by declining envoplakin titers at week 2 and 5 (envoplakin level was 7.41 and lowered to 5.47). This partial response is consistent with prior reports demonstrating that while tumor excision may decrease circulating autoantibody levels, irreversible end‐organ damage—particularly pulmonary—often continues to progress. Checking autoantibody titers may theoretically guide adjunctive treatment. However, in practice it takes 5–9 weeks to achieve complete serologic response after tumor resection [18]. Additionally, these send‐out tests often require days to weeks for results, and management of progressive, irreversible lung disease cannot be delayed while awaiting these labs [19]. Given the patient's presentation, a broad differential diagnosis was considered, including lymphoproliferative disorders such as lymphoma and plasma cell dyscrasias, which are well‐described associations with PNP. The patient underwent comprehensive oncologic evaluation without evidence of lymphoma or plasma cell neoplasm. In addition, evaluation for human herpesvirus‐8 (HHV‐8), which is classically associated with multicentric Castleman disease, was performed as part of the infectious and oncologic workup and was not identified, supporting a diagnosis of unicentric Castleman disease.

## Author Contributions

**Ayaan Sohail:** conceptualization, methodology, visualization, writing – original draft, writing – review and editing. **Jessica Evans:** data curation, resources, writing – review and editing. **Mika Tabata:** project administration, supervision, validation, writing – review and editing.

## Funding

The authors have nothing to report.

## Consent

Written informed consent was obtained from the patient for publication of this case report and accompanying images. A statement confirming this has been included in the manuscript.

## Conflicts of Interest

The authors declare no conflicts of interest.

## Data Availability

No datasets were generated or analyzed for this case report. All relevant clinical information has been deidentified and included within the manuscript. Additional patient‐level information is not publicly available to protect patient privacy and confidentiality.
